# Digital neuroplasticity modulation of the brain–immune axis: Translational strategies for neurodegenerative, psychiatric, and aging-related conditions

**DOI:** 10.4103/NRR.NRR-D-25-01728

**Published:** 2026-02-05

**Authors:** Merav Catalogna, Amir Amedi

**Affiliations:** Dina Recanati School of Medicine, Reichman University, Herzliya, Israel; Baruch Ivcher Institute for Brain, Cognition, and Technology, Baruch Ivcher School of Psychology, Reichman University, Herzliya, Israel

**The brain–immune axis:** The dynamic interplay between neural and immune systems is emerging as a fundamental regulator of cognitive processes, affective balance, and resilience to pathological challenges (Castellani et al., 2023). Although these systems detect and respond to distinct types of stimuli, their domains of perception and response substantially overlap. The nervous system primarily encodes physical and sensory stimuli, such as light, sound, temperature, and mechanical pressure, while the immune system detects molecular cues, including pathogens, cytokines, and endogenous danger signals. Together, both systems integrate biochemical, circadian, metabolic, and stress-related inputs to maintain adaptive and coordinated responses to internal and external perturbations (Leunig et al., 2025).

The brain communicates with the peripheral immune system through several interconnected pathways. The endocrine pathway involves the hypothalamic–pituitary–adrenal axis, which regulates cortisol via the adrenocorticotropic hormone to modulate immune responses. Additional hypothalamic hormones, including oxytocin, vasopressin, and growth hormone, further influence immune function. The autonomic nervous system exerts immunoregulatory control via both sympathetic and parasympathetic branches. Systemic and local sympathetic activity, including adrenal secretion of catecholamines (adrenaline and noradrenaline), broadly modulates immune cell function, whereas parasympathetic cholinergic signaling, primarily via acetylcholine, mediates anti-inflammatory effects. Sensory neurons detect peripheral threats and convey this information to the central nervous system (CNS), or locally regulate immune cells through neuropeptide release. Additionally, the meningeal lymphatic system facilitates the trafficking of immune cells and signaling molecules between the CNS and the periphery. Together, these pathways establish a coordinated, bidirectional network through which the nervous system integrates and regulates immune function (Schiller et al., 2021). These communication pathways promote direct interactions between peripheral immune cells and the CNS. In addition to humoral and cellular routes, vagal afferent fibers play a critical role in conveying peripheral inflammatory and immunological signals to central autonomic and limbic structures. Recent studies show that peripheral immune cells actively interact with the CNS. Particularly, monocytes and T cells can enter the CNS via transient blood–brain barrier disruptions or chemotactic signaling, releasing cytokines that modulate synaptic plasticity, neuronal excitability, and microglial activity (Leunig et al., 2025). Systemic insults, including environmental stressors or inflammatory challenges, can disrupt immune and metabolic homeostasis, impair brain function and behavior, and increase susceptibility to neurodegenerative diseases and psychiatric disorders. The immune system maintains neural and behavioral steady states by regulating neuroplasticity, neurogenesis, white matter integrity, and adaptive stress responses, highlighting the immune system’s central role in supporting cognition and emotional stability (Castellani et al., 2023).

**Neuroimmune modulation:** The brain-immune system integrates sensory inputs, including autonomic signaling through sympathetic and parasympathetic branches, and molecular mediators. Therefore, targeting these pathways offers a promising strategy to modulate neural and immune function. By leveraging these interconnected circuits, it may be possible to restore homeostasis, mitigate inflammation, and optimize brain function.

Brain plasticity refers to the brain’s intrinsic ability to dynamically adapt to environmental demands, physiological alterations, and experiences. It constitutes a fundamental mechanism underlying neuroplasticity, learning, and functional recovery that persists throughout life. These adaptive processes are expressed through modifications in synaptic efficacy, reorganization of neural network activity, and, in certain contexts, structural regeneration or neurogenesis (Pascual-Leone et al., 2005). Several mechanisms can trigger brain plasticity in response to functional decline. Pharmacological agents that enhance neurotransmission, neurotrophic signaling, or metabolic support can facilitate synaptic remodeling in neurodegenerative and psychiatric disorders. Selective serotonin reuptake inhibitors, in particular, and ketamine may increase brain-derived neurotrophic factor expression, promoting neuronal integrity and synaptic plasticity (Gazerani, 2025). Neuromodulation techniques, such as neurofeedback, repetitive transcranial magnetic stimulation, and transcranial direct current stimulation, can also modulate dysfunctional networks and promote adaptive reorganization. These methods enhance neuroplasticity and functional improvement by influencing neurotransmitter systems and receptor activity (Pascual-Leone et al., 2005; Gazerani, 2025).

Physical and cognitive interventions, such as cognitive training, enriched environments, and psychological therapies, have also been shown to influence brain structure and function across healthy aging and various neurological and psychiatric conditions. Engaging in consistent physical activity enhances brain-derived neurotrophic factor levels, potentially improving cognitive performance and emotional resilience (Gazerani, 2025). Lately, experiments employing tailored cognitive neuroscience techniques and multisensory protocols highlight adaptive and compensatory neuroplastic mechanisms in adult brains (Pascual-Leone et al., 2005; Amedi et al., 2024). Investigations of sensory deprivation, in congenital or late-onset blindness, as well as in blindfolding of sighted individuals, demonstrate that alternative sensory pathways can recruit cortical regions that are typically dormant. For example, visual-to-auditory training can engage the occipital cortex via non-visual modalities, thereby strengthening previously inhibited or weak connections and networks. These findings illustrate the brain’s ability to reallocate neural resources in response to sensory experiences and targeted interventions (Pascual-Leone et al., 2005; Amedi et al., 2024).

Many of these interventions have also associated with changes in immune system function. Psychosocial therapies, including cognitive behavioral therapy and multicomponent psychotherapies, were found to reduce proinflammatory cytokines, with effects persisting for months (Shields et al., 2020). Given bidirectional brain-immune communication, interventions targeting one system can reciprocally influence the other. Thus, combining complementary intervention types may produce complementary effects, simultaneously enhancing neuroplasticity and immune regulation. For example, pharmacological agents that enhance neurotransmission and neurotrophic signaling can optimize the neurochemical environment to support learning-related brain changes, while cognitive or motor training engages neural and autonomic networks that regulate immune function. Together, these mechanisms frame the conceptual basis for integrative therapeutic models that leverage coordinated brain-body interactions to support functional recovery.

**Figure 1:**
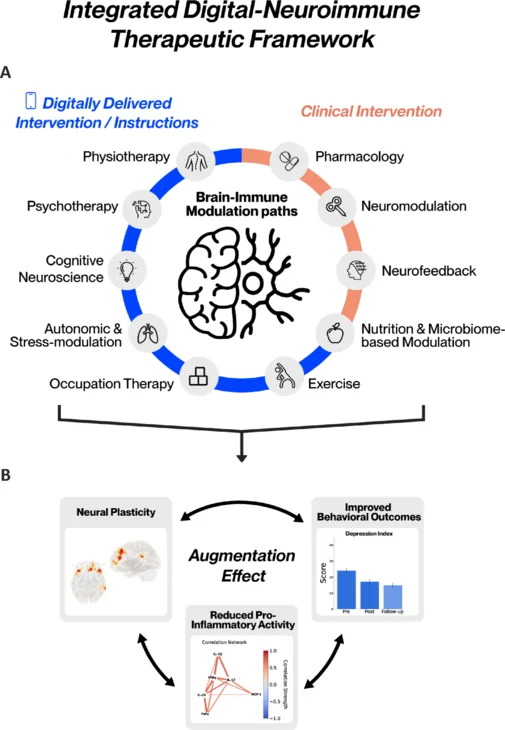
Integrated digital-neuroimmune therapeutic framework. (A) A conceptual framework illustrating brain-immune modulation via digitally delivered modules and clinical strategies. These modules include psychotherapeutic, cognitive training, autonomic, physical, nutritional, and rehabilitation components that may individually or collectively improve neural, immune, and behavioral outcomes. Combined with pharmacological or neuromodulation therapies, such digital interventions can enhance treatment efficacy, supporting an integrative neurobehavioral and neuroimmune approach. (B) An example of the effect of RMPY-008. By combining psychotherapy and cognitive-neuroscience components, the intervention was associated with a downregulation of inflammatory markers alongside measurable improvements in affective symptoms and psychological coping. At the neural level, changes in fronto-limbic connectivity were observed and showed significant associations with both immunological and psychological outcomes. Adapted from Catalogna et al. (2025a). IFN-γ: Interferon gamma; IL-12: interleukin 12; IL-17: interleukin 17; IL-23: interleukin 23; MCP-1: monocyte chemoattractant protein-1; TNF-α: tumor necrosis factor-alpha.

**Digital and multimodal interventions:** Multimodal non-pharmacological interventions are recommended by clinical practice guidelines for the primary care management of neurodegenerative and psychiatric disorders. Nevertheless, their accessibility and integration into standard clinical practice remain limited, where principal barriers include geographic, financial, logistical, and public health educational constraints. Digital therapy offers an opportunity to bridge this gap, both as an adjunct to pharmacological and behavioral therapies and potentially as a stand-alone treatment. However, digital therapy has not yet been widely integrated into standard clinical practice, and patient adherence requires systematic evaluation. A therapeutic framework designed to advance the clinical integration of digital therapy through brain-immune modulation may incorporate digitally delivered modules adapted from different neuroimmune modulation strategies. These modules can combine psychotherapeutic components, cognitive neuroscience-based training, autonomic regulation, physical activity, nutrition, and rehabilitation strategies, which can individually or collectively contribute to therapeutic effects across neural, immune, and behavioral systems. In conjunction with pharmacological or neuromodulation techniques, such digital interventions may augment and enhance treatment efficacy, providing a translational framework for optimized medical care (**Figure 1A**). These mechanisms give rise to a continuous feedback loop, mediated by neuroimmune signaling pathways that dynamically adjust through sustained digital engagement and ongoing therapeutic modulation.

Building on this framework, we evaluated a potential triple association between digital intervention-induced changes in neural plasticity, peripheral immune activity, and behavioral outcomes. In a recent study, we demonstrated that a 3-week digital daily training protocol delivered via a smartphone application produced measurable improvements across those domains in 103 individuals experiencing subjective cognitive decline and elevated anxiety in comparison to a waitlist-control group (Catalogna et al., 2025a). Subjective cognitive decline was chosen as a model of early neurodegeneration, where affective disturbances can accelerate cognitive decline and promote a pro-inflammatory profile. The digital intervention combined evidence-based psychotherapeutic approaches from well-established disciplines, such as cognitive behavioral therapy, mindfulness-based programs (mindfulness-based stress reduction, mindfulness-based cognitive therapy), acceptance and commitment therapy, and psychoeducation, which have all previously demonstrated effectiveness across various mental health conditions. These were combined with sensorimotor-deprivation tasks, which can enhance neural plasticity, alongside exercises designed to enhance parasympathetic activity, such as progressive muscle relaxation and controlled breathing techniques.

The study demonstrated that daily engagement with the RMPY-008 app (Remepy Inc., New York, NY, USA) resulted in significant reductions in depression and situational anxiety, alongside enhanced resilience and wellbeing. These effects were sustained or augmented throughout a 3-week phase of less frequent intervention. Peripheral blood analyses indicated a marked decrease in pro-inflammatory cytokine levels following the intervention (**Figure 1B**), and resting-state functional magnetic resonance imaging demonstrated that these psychological and immunological changes were associated with altered functional connectivity in the insula and frontal regions within the default mode, central executive, and salience networks. These findings align with Menon and Uddin’s model identifying the insula as a core integrator mediating dynamic interactions between these large-scale brain networks. From a brain-immune perspective, the observed association between insula-centered neural changes and reductions in peripheral immune activity could reflect mechanisms similar to the bidirectional regulatory loop described by Rolls (2023). Finally, consistent with this integrative role, Zeharia et al. (2015) identified a somatotopic body representation (body homunculus) within the insula, providing additional neuroanatomical basis for body-mind interactions. Supporting these results, our earlier pilot study reported similar changes in mood and large-scale network dynamics, along with reduced salivary interleukin-18 levels, in a smaller cohort undergoing a comparable app-based intervention (Catalogna et al., 2025b). Importantly, while these results demonstrate meaningful associations between digital intervention, psychological outcomes, and neuroimmune markers, establishing mechanistic causality requires further investigation. Future studies should incorporate pre-registered mediation analyses to evaluate whether neuroplasticity and autonomic changes mediate immune effects. Moreover, experimental perturbations employing dose-response analyses and temporal precedence testing will be critical to confirm the magnitude and directionality of these relationships. Special attention should be given to controlling circadian timing of endocrine and immune markers, particularly rhythmic hormones such as cortisol, and considering hypothalamic-pituitary-adrenal-immune models to strengthen mechanistic interpretation.

Finally, the reduction in pro-inflammatory cytokines may have significant consequences not only for emotional health but also for mitigating the advancement of neurodegenerative pathology. Neuroinflammation and systemic inflammation are recognized contributors to the onset and progression of neurodegenerative diseases and age-related cognitive decline, including mild cognitive impairment and Alzheimer’s disease (Castellani et al., 2023). Additionally, studies have highlighted the significance of the immune system for lifelong brain maintenance (Castellani et al., 2023). Together, this relationship and the co-modification of brain plasticity, immune signaling, and psychological processes suggest a route toward systemic biological modulation via digital behavioral pathways.

**Conclusions and future directions:** This perspective highlights the promise of digital therapeutics as both stand-alone and combined digital-drug treatment regimens, while also pointing toward several critical avenues for future research. First, as regulatory bodies increasingly recognize and approve prescribing digital therapeutics for psychiatric and neurological conditions, rigorous mechanistic studies will be essential to define the biological implications of their effects and to establish clinical, technical, and regulatory standards (Ferrante et al., 2024). Second, the broader relevance of this approach extends well beyond subjective cognitive decline. Digital interventions can incorporate any validated therapeutic component into a unified digital protocol, allowing this framework to adapt to a wide range of clinical contexts (e.g., neurological, psychiatric, cardiovascular, metabolic, autoimmune, and oncological diseases). Finally, realizing the vision of precision neuroimmune therapeutics will require combining scalable digital interventions with biomarker-based (neural, immune markers) monitoring and pharmacological treatments, enabling adaptive tailoring to an individual’s behavioral, neural, and immune responses. Artificial intelligence may serve as a key driver of this precision, supporting treatment personalization, adherence, and patient engagement through features such as digital care coordinators and conversational support tools.

Importantly, while digital therapeutics hold significant promise, their successful translation into scalable clinical applications demands careful attention to ethical and regulatory standards. Hybrid digital interventions inherently involve processing sensitive personal data, requiring strict data minimization, encryption, transparency in personalization algorithms, mitigation of artificial intelligence bias, and user control over data sharing. Given the evolving nature of software-based interventions, rigorous evidence standards, including protocol registration, pre-specified endpoints, documentation of software versioning, and independent validation, are essential to maintain scientific and clinical trust. Lastly, equitable access, clinical safety monitoring, and clear pathways for integration and reimbursement are critical to ensure sustainable adoption within healthcare systems (Pham, 2025). Integrating these considerations will be critical to advancing digital therapeutics that are both efficacious and ethically responsible.

In conclusion, while fully considering ethical and regulatory principles, this integrated framework provides a promising avenue for investigating the bidirectional interactions between the brain, the immune system, and behavior, while also demonstrating digital therapeutics’ potential to expand treatment beyond traditional clinical frameworks.
